# Simplified Calculation Model and Experimental Study of Latticed Concrete-Gypsum Composite Panels

**DOI:** 10.3390/ma8105375

**Published:** 2015-10-27

**Authors:** Nan Jiang, Shaochun Ma

**Affiliations:** 1 School of Civil Engineering, Tianjin University, Tianjin 300072, China; jiangnan@tju.edu.cn; 2 Key Laboratory of Coastal Civil Engineering Structure and Safety (Tianjin University), Ministry of Education, Tianjin 300072, China

**Keywords:** lattice, composite, equivalent constants, simplified calculation, experiment, method

## Abstract

In order to address the performance complexity of the various constituent materials of (dense-column) latticed concrete-gypsum composite panels and the difficulty in the determination of the various elastic constants, this paper presented a detailed structural analysis of the (dense-column) latticed concrete-gypsum composite panel and proposed a feasible technical solution to simplified calculation. In conformity with mechanical rules, a typical panel element was selected and divided into two homogenous composite sub-elements and a secondary homogenous element, respectively for solution, thus establishing an equivalence of the composite panel to a simple homogenous panel and obtaining the effective formulas for calculating the various elastic constants. Finally, the calculation results and the experimental results were compared, which revealed that the calculation method was correct and reliable and could meet the calculation needs of practical engineering and provide a theoretical basis for simplified calculation for studies on composite panel elements and structures as well as a reference for calculations of other panels.

## 1. Introduction

Gypsum is one of the three major cementing materials that are widely used in the fields of building materials, food, precision casting, models and molds, medicine, paper, and paint fillers. As early as 2000~3000 BC, humans attempted using gypsum as a cementing material to build the famous Egyptian pyramids, ancient Roman architecture, Mogao Caves at Dunhuang, *etc*. Since Louis XIV, known as France’s Sun King, issued a decree in 1667, calcined gypsum (building gypsum) started to be widely used in the building industry. Building gypsum is primarily comprised of beta-calcium sulfate hemihydrate (β-CaSO_4_•1/2H_2_O), whose content should be not lower than 60.0% [1]. Building gypsum expands by 0.05%~0.15% after hardening, with a compressive strength of 5~10 MPa, a flexural strength of 2.5 MPa, and a density of 13.0~14.5 kN/m^3^ [2]. In the construction industry, gypsum is generally used for the cementing material, decorative gypsum boards, reliefs, roman columns, *etc.*; non-load-bearing partitions such as hollow gypsum boards and dense-column composite panels with low load-bearing capacity; and load-bearing, anti-seismic walls with high load-bearing capacity such as the (dense-column) latticed concrete-gypsum composite panel studied in this paper. The (dense-column) latticed concrete-gypsum composite panel is a composite of environmentally-friendly gypsum, fiberglass, concrete, and steel bars that has excellent seismic performance, is lighter than ordinary shear walls, and contains non-toxic, harmless, pollution-free gypsum that can adjust indoor humidity automatically with good thermal and sound insulation and fire resistance [3]. The development and wide use of (dense-column) latticed concrete-gypsum composite panels have promoted the innovation in new wall materials and the development of green, energy-saving walls. This kind of composite panel makes full use of the advantages of its constitute materials. The tensile fiber increases the gypsum board’s ability to withstand forces. The gypsum board performs the (dense-column) latticed concrete-gypsum composite panel’s maintenance and thermal and sound insulation functions while serving as a stressed member [4]. The excellent material properties of concrete enable it to bear heavy loads. As indicated above, the composite panel reflects an effective composite of the functional and mechanical aspects of the various constituent materials. Scholars in Australia, China, India, the United States, and Italy have conducted extensive experimental studies on composite panels [5,6,7,8,9]. However, few achievements have been made in simplifying calculations that are difficult to operate and implement. A great number of gypsum partitions that exist in the gypsum board of a (dense-column) latticed concrete-gypsum composite panel partition the hollow gypsum board into several horizontal and vertical gypsum cavities. After concrete pouring, a lattice of invisible beam-invisible column forms, making simplified calculation difficult and challenging. The greatest difficulty confronting the simplified calculation of composite panels lies in the determination of basic elastic constants due to the complexity of the composite [10], directly leading to the difficulty in engineering software modeling, a great number of computing elements, a great amount of calculation work, and a failure to make effective calculation.

The (dense-column) latticed concrete gypsum composite panel in this study primarily consisted of the porous gypsum board with cavities and the invisible beam-invisible column lattice of concrete. The composite panel with this structure not only has a high bearing capacity, but also facilitates the arrangement of steel bars and the processing of nodes. In order to find a feasible simplified calculation model for the (dense-column) latticed concrete-gypsum composite panel, a technical solution was established. A typical element of this composite panel was selected to establish an effective calculation model. Through a series of theoretical deviations, a feasible simplified calculation model was obtained. Finally, the reliability of the simplified calculation model was validated by comparing the seismic experimental results and the calculation results of the composite panel.

## 2. Technical Solution to Simplified Calculation

**Figure 1:**
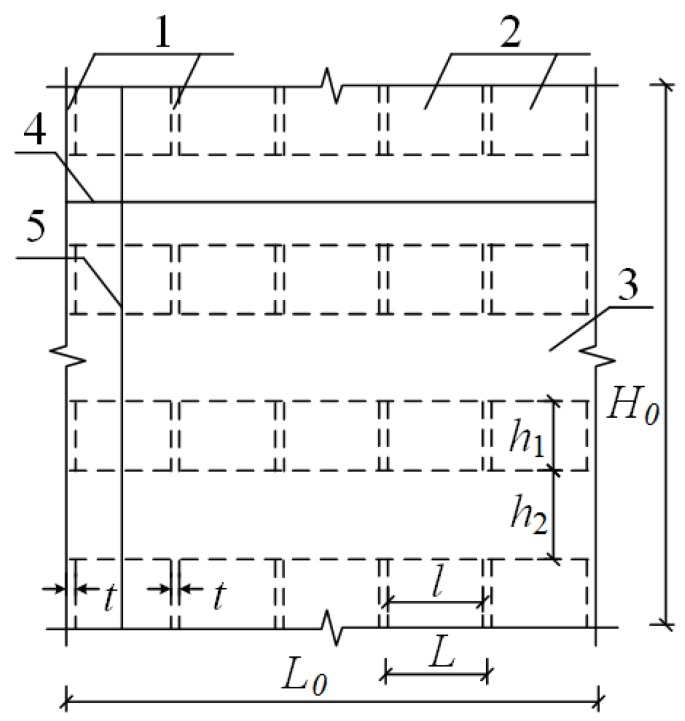
Perspective of the gypsum board.

As shown in Figure 1, (1) is a gypsum partition; (2) is a cavity formed vertically by gypsum partitions; (3) is a cavity formed horizontally by gypsum partitions; (4) is an invisible concrete beam formed at the horizontal cavity after concrete pouring; and (5) is an invisible concrete column formed at the vertical cavity after concrete pouring. The invisible beams and invisible columns form an entire beam-column lattice structure. The porous gypsum board primarily consists of two gypsum side panels and *n* gypsum partitions (length × width × height = 94 mm × 20 mm × 160 mm). Gypsum partitions are reasonably arranged between the two gypsum side panels to form many horizontal gypsum cavities (length × width = 220 mm × 94 mm) and vertical gypsum cavities (length × width = 230 mm × 94 mm) in the entire gypsum board. These cavities can be used as the templates for concrete pouring.

**Figure 2:**
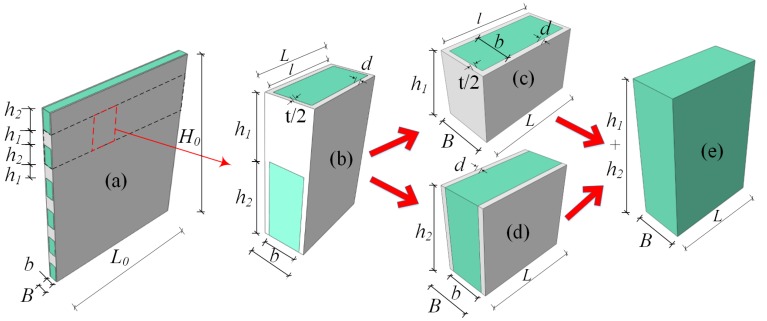
Technical solution to the calculation of elastic constants: (**a**) the (dense-column) latticed concrete gypsum composite panel; (**b**) a typical homogenous composite element; (**c**) the homogenous composite sub-element I; (**d**) the homogenous composite sub-element II; and (**e**) a secondary homogenous composite.

The above introduction revealed that a (dense-column) latticed concrete gypsum composite panel has a complex structure, and the performance of its constituent materials differs greatly, which determines the special complexity of its mechanical properties and makes it difficult to calculate. Therefore, it is hard to obtain the accurate mechanical results of composite panels. Simple homogenization has been incompetent, and a new way is required. The aim of this study is to enable the calculation results to meet engineering requirements, simplify the calculation method as much as possible and significantly reduce the amount of calculations and time required for calculations. The specific technical solution is shown in Figure 2. From the perspective of the mechanics of the composite, a typical homogenous composite element of the (dense-column) latticed concrete gypsum composite panel was selected, as shown in Figure 2b. The homogenous composite element was then divided into two homogeneous composite sub-elements, as shown in Figure 2c,d. These two homogenous composite sub-elements were rendered homogenously equivalent, respectively. Then the two equivalent homogenous composite sub-elements were used to constitute a secondary homogenous element, as shown in Figure 2e. The secondary homogeneous element was used to substitute the original homogenous composite element, and the basic dimensions and mechanical properties of the composite panel were maintained unchanged before and after equivalent treatment, thus realizing the transformation from a complex (dense-column) latticed concrete gypsum composite panel to a simple homogenous panel, and greatly reducing the difficulty in calculation. The valuable results obtained finally will be of great practical significance to the in-depth studies on the mechanical properties of (dense-column) latticed concrete gypsum composite panel elements and structures.

## 3. Theoretical Derivations of Elastic Constants

### 3.1. Basic Assumptions

(1) The gypsum board, fiberglass, and concrete were all isotropic materials and linearly elastic; (2) the fiberglass was evenly distributed in the gypsum board, and the various materials of the composite panel bonded firmly; and (3) the effects of the residual stresses and strains in the various materials of the composite panel were ignored [11].

With great in-plane stiffness [12], floors can constrain the out-of-plane deformation of composite panels. Therefore, only the in-plane action needs to be considered for a composite panel. In other words, a composite panel can be treated as a plane. In the following, formula derivation was conducted for the simplified calculation of composite panels.

### 3.2. Calculation of Elastic Constants

*E_c_*, *E_g_*, *G_c_*, and *G_g_* represent the elastic modulus and shear modulus of the concrete and gypsum board, respectively (obtained through material tests). The equivalent elastic modulus of the homogenous composite sub-element I in the *X* or *Y* direction is *E_Ix_* or *E_Iy_*, and its equivalent shear modulus and Poisson’s ratio in the *XY* plane are *G_Ixy_* and *v_Ixy_*, respectively. Similarly, those of the homogenous composite sub-element are *E_IIx_*, *E_IIy_*, *G_IIxy_*, and *v_IIxy_*, respectively, and those of the secondary homogenous element are *E_x_*, *E_y_*, *G_xy_*, and *v_xy_*, respectively. Due to symmetry, 1/4 of the two homogenous composite sub-elements were analyzed, and the entire secondary homogenous element was analyzed, as detailed below.

#### 3.2.1. Homogenous Composite Sub-Element I

**Figure 3:**
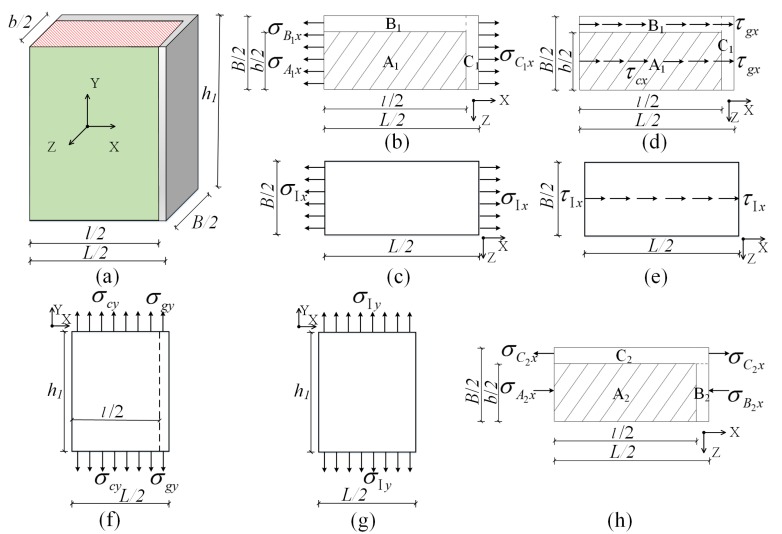
Models of Homogenous Composite Sub-element I: (**a**) 1/4 of the three-dimensional homogenous composite sub-element I; (**b**) the calculation model for solving *E_Ix_*; (**c**) equivalent model for solving *E_Ix_*; (**d**) the calculation model for solving *G_Ixy_*; (**e**) equivalent model for solving *G_Ixy_*; (**f**) the calculation model for solving *E_Iy_*; (**g**) equivalent model for solving *E_Iy_*; and (**h**) the calculation model for solving *v_Ixy_*.

Figure 3a shows 1/4 of the three-dimensional homogenous composite sub-element I. For ease of derivation, the force diagrams of the homogenous composite sub-element in the *X*–*Z* and *X*–*Y* planes were given, respectively. Figure 3b,c are the calculation model and equivalent model for solving *E_Ix_*; Figure 3d,e are the calculation model and equivalent model for solving *G_Ixy_*; Figure 3f,g are the calculation model and equivalent model for solving *E_Iy_*, and Figure 3h is the calculation model for solving *v_Ixy_*.

(1) Elastic modulus *E_Ix_*: As shown in Figure 3b,c, the planar calculation model of the homogenous composite sub-element I was divided into the three calculation domains, A_1_, B_1_, and C_1_. B_1_ and C_1_ are the gypsum boards, and A_1_ is concrete. In the *X* direction, the sum of the normal stresses of the calculation model of the composite panel and that of the equivalent model should be equal under the same loading [13].

(1) $${\text{σ}}_{Ix}={\text{σ}}_{C_{1}x},{\text{σ}}_{Ix}h_{1}B/2={\text{σ}}_{A_{1}x}h_{1}b/2+{\text{σ}}_{B_{1}x}h_{1}(B/2-b/2)$$

where σ*_A_1___x_*, σ*_B_1___x_*, σ*_C_1___x_*, and σ*_Ix_* are the stresses of concrete, gypsum board (namely domain B_1_ and C_1_), and the equivalent model in the X direction, respectively. ε*_Ix_* is the strain of the equivalent element I in the X direction. The strain in the homogenous composite sub-element I in the *X* direction ε*_Ix_* should be the same before and after equivalent treatment.

(2) $${\text{σ}}_{Ix}/E_{Ix}={\text{σ}}_{A_{1}x}/E_{c}={\text{σ}}_{B_{1}x}/E_{g}={\text{ε}}_{Ix}$$

Let b/B=λ. Equation (2) was substituted into Equation (1) to obtain

(3) $$E_{Ix}=\text{λ}E_{c}+(1-\text{λ})E_{g}$$

(2) Elastic modulus *E_Iy_*: As shown in Figure 3f,g, σ*_cy_*, σ*_gy_*, and σ*_Iy_* are the stresses of concrete, the gypsum board, and the equivalent model in the Y direction, respectively, and ε*_Iy_* is the strain of the equivalent element I in the Y direction. Similarly,

(4) $${\text{σ}}_{Iy}BL/4={\text{σ}}_{c}bl/4+{\text{σ}}_{g}(BL-bl)/4$$

(5) $${\text{σ}}_{Iy}/E_{Iy}={\text{σ}}_{c}/E_{c}={\text{σ}}_{g}/E_{g}={\text{ε}}_{Iy}$$

Let l/L=β. Equation (5) and λ were substituted into Equation (4) to obtain

(6) $$E_{Iy}=\text{λβ}E_{c}+(1-\text{λβ})E_{g}$$

(3) Shear modulus *G_Ixy_*: τ_cx_, τ_gx_ and τ*_I_*_x_ are the shear stresses of concrete, the gypsum board (namely domain B_1_ and C_1_), and the equivalent model in the *X* direction. As shown in Figure 3d,e, the similar method was used to obtain

(7) $$G_{Ixy}=\text{λβ}G_{c}+(1-\text{λβ})G_{g}$$

(4) Poisson’s ratio *v_Ixy_*: The planar calculation model of the homogenous composite sub-element I was divided into the three calculation domains A_2_, B_2_, and C_2_. B_2_ and C_2_ are the gypsum boards, and A_2_ is concrete. Where σ*_A_2_x_*, σ_*B_2_x*_, and σ_*C_2_x*_ are the stresses of concrete and gypsum board (namely domain B_2_ and C_2_) in the X direction. As shown in Figure 3h, the strain difference between the combination of A_2_ and B_2_ and C_2_ in the *X* direction is $0.5l(\nu_{g}-\nu_{c}){\text{ε}}_{Iy}$.

According to the conditions for equilibrium of forces,

(8) $$(B-b){\text{σ}}_{C_{2}x}=b{\text{σ}}_{B_{2}x}$$

Since

(9) $${\text{ε}}_{Ix}=\nu_{Ixy}{\text{ε}}_{Iy}=\nu_{g}{\text{ε}}_{Iy}-{\text{σ}}_{C_{2}x}/E_{g}$$

The displacements or strains on the left and right sides were required to satisfy the coordination:

(10) $${\text{σ}}_{C_{2}x}\text{L}/2E_{g}+{\text{σ}}_{B_{2}x}l/2E_{\text{c}}+{\text{σ}}_{B_{2}x}(\text{L}-l)/2E_{g}=(\nu_{g}-\nu_{c}){\text{ε}}_{Iy}l/2$$

Let $E_{\text{g}}/E_{\text{c}}=\text{α}$. λ and β were substituted to obtain

(11) $$\nu_{Ixy}=\nu_{g}-\text{βλ}(\nu_{g}-\nu_{c})/[\text{λ}+(1-\text{λ})(1-\text{β}+\text{αβ})]$$

#### 3.2.2. Homogenous Composite Sub-Element II

**Figure 4:**
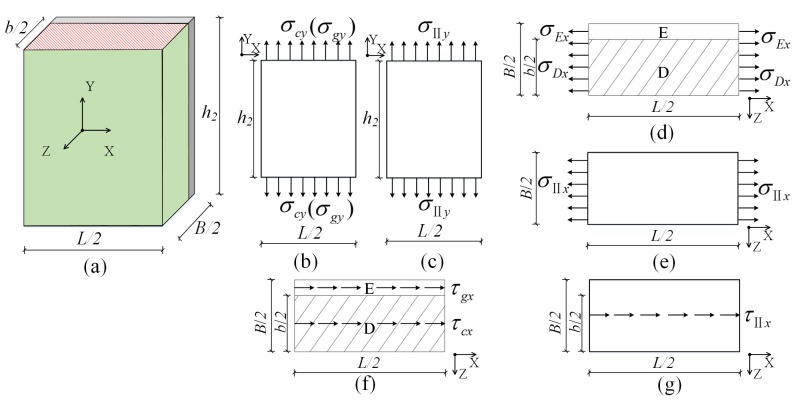
Models of Homogenous Composite Sub-element II: (**a**) 1/4 of the three-dimensional homogenous composite sub-element II; (**b**) the calculation model for solving *E_IIy_*; (**c**) equivalent model for solving *E_IIy_*; (**d**) the calculation model for solving *E_IIx_*; (**e**) equivalent model for solving *E_IIx_*; (**f**) the calculation model for solving *G_IIxy_*; and (**g**) equivalent model for solving *G_IIxy_*.

(1) Elastic modulus *E_IIx_*: As shown in Figure 4d,e, similarly,

(12) $${\text{σ}}_{IIx}h_{2}B/2={\text{σ}}_{Dx}h_{2}\text{​}b\text{​}/2+{\text{σ}}_{Ex}h_{2}(B-b)/2,{\text{σ}}_{II\text{​}x}/E_{IIx}={\text{σ}}_{Dx}/E_{c}={\text{σ}}_{Ex}/E_{g}={\text{ε}}_{IIx}$$

σ*_IIy_* is the stress of the equivalent model in the *Y* direction. σ*_Dx_*, σ*_Ex_*, and σ*_IIx_* are the stresses of concrete, gypsum board, and the equivalent model in the *X* direction. ε*_IIx_* and ε*_IIy_* are the strains of the equivalent model in the *X* and *Y* directions.

Therefore,

(13) $$E_{IIx}=\text{λ}E_{c}+(1-\text{λ})E_{g}$$

(2) Elastic modulus *E_IIy_*: As shown in Figure 4b,c, similarly,

(14) $${\text{σ}}_{II\text{​}y}BL/4={\text{σ}}_{cy}bL\text{​}/4+{\text{σ}}_{gy}\left(B-b\right)L/4,{\text{σ}}_{II\text{​}y}/E_{IIy}={\text{σ}}_{cy}/E_{c}={\text{σ}}_{gy}/E_{g}={\text{ε}}_{IIy}$$

Therefore,

(15) $$E_{IIy}=\text{λ}E_{c}+(1-\text{λ})E_{g}$$

(3) Shear modulus *G_IIxy_*: τ_cx_, τ_gx_ and τ*_II_*_x_ are the shear stresses of concrete (namely domain D), the gypsum board (namely domain E), and the equivalent model in the *X* direction. As shown in Figure 4f,g, the same method was used to obtain

(16) $$G_{IIxy}=\text{λ}G_{c}+(1-\text{λ})G_{g}$$

(4) Poisson’s ratio *v_IIxy_*: As shown in Figure 4d, when the composite panel is stretched evenly in the *X* direction, its contraction strain is equal to the sum of the contraction strains of gypsum and concrete in the *Z* direction, namely

(17) $${\text{ε}}_{IIz}=\nu_{IIxz}{\text{ε}}_{IIx}B/2=[\nu_{c}b/2+\nu_{g}(B-b)/2]{\text{ε}}_{IIx}$$

Similarly, *λ* was substituted to obtain

(18) $$\nu_{IIxy}=\nu_{IIxz}=\nu_{IIyz}=\text{λ}\nu_{c}+（1-\text{λ}）\nu_{g}$$

#### 3.2.3. Secondary Homogenous Element

**Figure 5:**
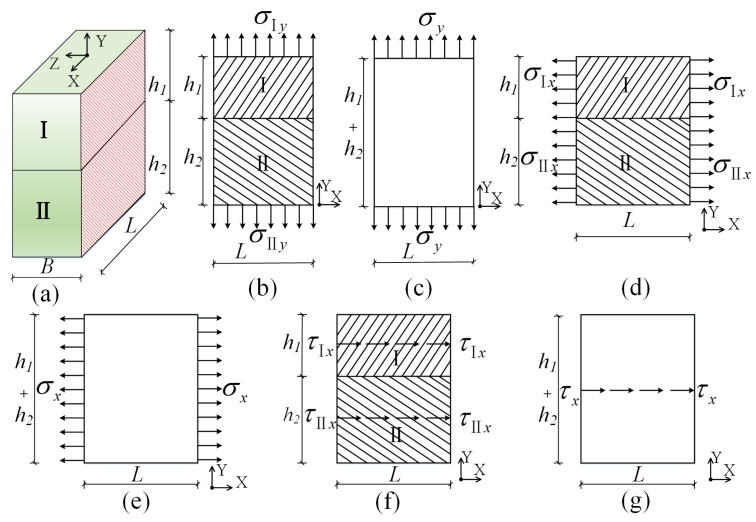
Models of Secondary Homogenous Element: (**a**) the three-dimensional secondary homogenous element; (**b**) the calculation model for solving *E_y_*; (**c**) equivalent model for solving *E_y_*; (**d**) the calculation model for solving *E_x_*; and (**e**) equivalent model for solving *E_x_*.

As shown in Figure 5a, the secondary homogenous element consisted of the homogenous composite sub-elements I and II. σ*_x_*, σ*_y_*, τ*_x_*, and τ*_y_* and are the normal stresses and shear stresses of the secondary homogenous element, respectively.

(1) Elastic modulus *E_y_*: As shown in Figure 5b,c, the sums of the normal strains of the two models under the same loading should be equal.

(19) $${\text{ε}}_{y}(h_{1}+h_{2})=h_{1}{\text{ε}}_{Iy}+h_{2}{\text{ε}}_{IIy}$$

The stresses in the *Y* direction are equal, namely

(20) $$E_{Iy}{\text{ε}}_{Iy}=E_{IIy}{\text{ε}}_{IIy}=E_{y}{\text{ε}}_{y}={\text{σ}}_{y}$$

Let $h_{1}/h_{2}=\text{ζ}$, which was substituted into Equation (19) to obtain

(21) $$E_{y}=(1+\text{ζ})E_{Iy}E_{IIy}/(E_{Iy}+\text{ζ}E_{IIy})$$

(2) Elastic modulus *E_x_*: As shown in Figure 5d,e, the method for calculating *E_IIx_* was used.

Therefore,

(22) $$E_{x}\text{=}(\text{ζ}E_{Ix}+E_{IIx})/(1+\text{ζ})$$

(3) Shear modulus *G_xy_*: The calculation model and equivalent model are shown in Figure 5f,g. The method for calculating *G_IIxy_* was used.

Therefore,

(23) $$G_{xy}\text{=}(\text{ζ}G_{Ixy}+G_{IIxy})/(1+\text{ζ})$$

(4) Poisson’s ratio *v_xy_*: The method for calculating *v_IIxy_* was used to obtain

(24) $$\nu_{xy}=(\nu_{Ixy}\text{ζ}+\nu_{IIxy})/(1+\text{ζ})$$

## 4. Experiment

### 4.1. Sample Design and Loading System

**Figure 6:**
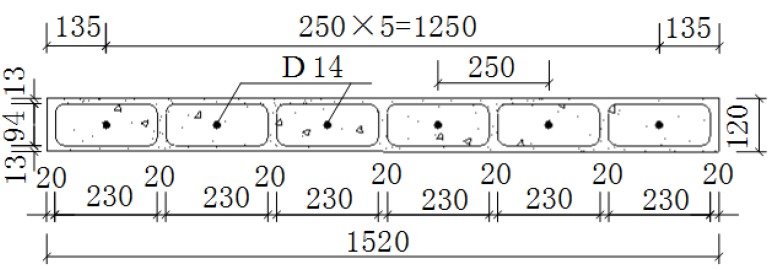
Plane of the composite panel.

**Figure 7:**
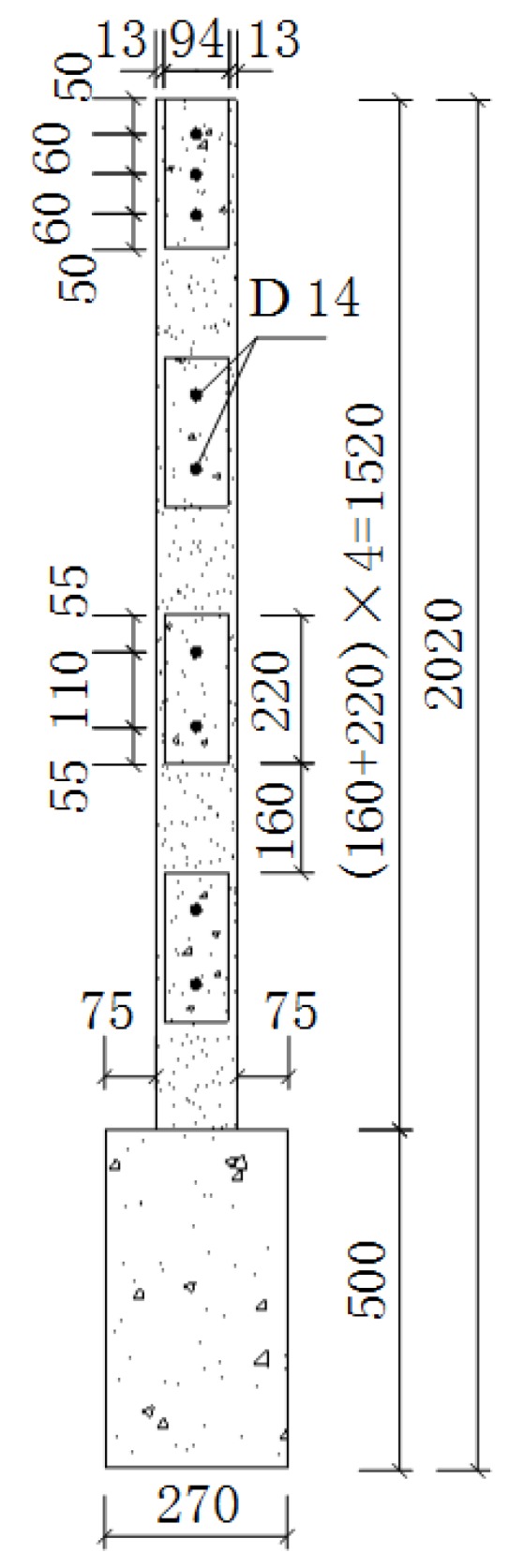
Elevation of the composite panel.

**Figure 8:**
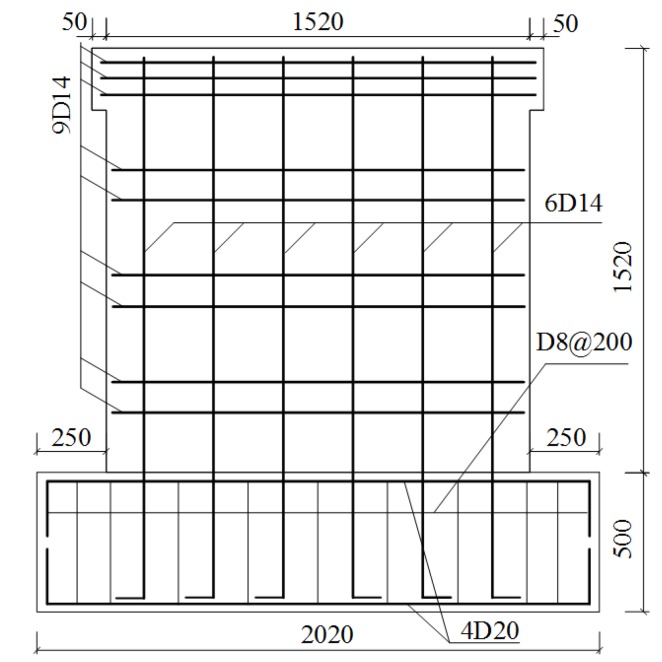
Reinforcement of the composite panel.

Three samples (1520 mm × 1520 mm × 120 mm) were designed for the seismic experiment on the composite panel in this study. They were numbered Q-1, Q-2, and Q-3, respectively. The plan and elevation of each sample are shown in Figure 6 and Figure 7, respectively. The steel bars arranged in the composite panel were HRB400 deformed steel bars with a diameter of 14 mm, as shown in Figure 8. The experimental results of the materials of the composite panel are shown subsequently. The compressive strength and elastic modulus of the gypsum board were 5.52 MPa and 4350 MPa, respectively. The compressive strength and elastic modulus of the concrete were 24.63 MPa and 2.72 × 10^4^ MPa, respectively. The tensile strength of the HRB400 steel bars was 654.00 MPa (D8), 669.45 MPa (D14), and 676.92 MPa (D20), respectively [14].

**Figure 9:**
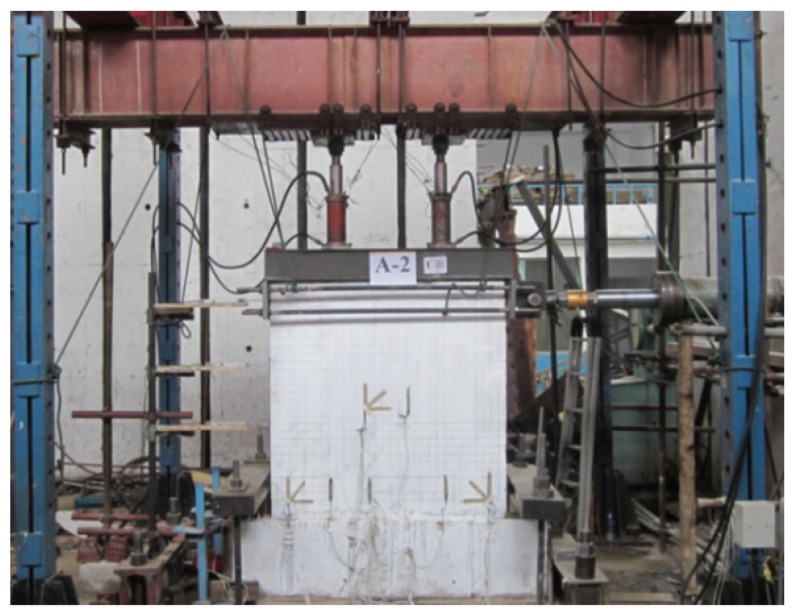
Loading devices at the experimental site figure.

The loading devices at the experimental site are shown in Figure 9 and consisted of a horizontal loading device and a vertical loading device [15,16,17]. The horizontal cyclic load was imposed by a 1000 kN hydraulic jack whose back was secured to the reaction wall on the right side. The vertical load was imposed by two 500 kN hydraulic jacks, which were secured to the sliding supports above. The sliding supports were secured to the reaction steel beam above. In order to accurately simulate the action of vertical loading on the composite panel under earthquake events in the process of horizontal cyclic loading, the vertical jacks were enabled to move synchronously with the sample with the aid of a sliding car, which ensured that the vertical load always acted on the center of the top of the sample. A rigid distributive beam was placed between the two 500 kN hydraulic jacks and the composite panel in order to generate uniform compressive stress on the top surface of the composite panel in the loading process. A displacement meter was placed at the side of the sample to acquire the displacement of the sample with the load. A dial indicator was placed at the end of the fixed beam at the bottom of the sample to detect whether the sample in its entirety moved in the experimental process in order to eliminate the displacement error generated by the fixed beam during subsequent data processing. Resistance strain gauges were installed at the key positions of the steel bar, invisible concrete beam, invisible concrete column, and outside gypsum board of the panel sample to measure the strains in the various materials of the sample. For example, the 45° strain gauge on the sample could be used to measure the shear deformation of the panel. The data collected by the displacement meter, dial indicator, and various resistance strain gauges, and the sensors attached to the jacks were stored in a computer for real-time collection.

The quasi-static test method defined in the *Specification of Testing Methods for Earthquake Resistant Building* (JGJ101-96) was performed [18]. First, a constant vertical load was imposed, and then a horizontal cyclic load was imposed. When the composite panel was in the elastic stage, a load-controlled loading system was adopted. When the composite panel was in the elastic-plastic stage, a load- and displacement-controlled loading system was used until the sample fractured.

### 4.2. Main Experimental Results

#### 4.2.1. Experimental Process and Fracture Phenomena

**Figure 10:**
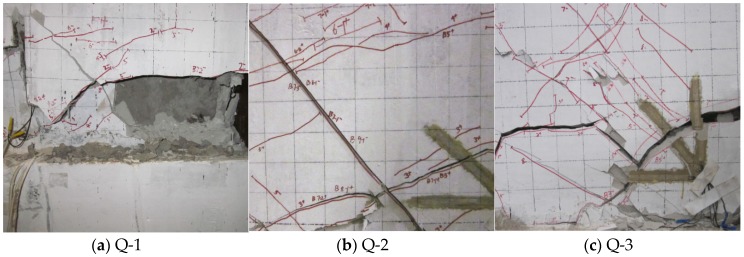
Fracture of samples (**a**) Q-1; (**b**) Q-2; (**c**) Q-3.

The experiment was conducted to study the bearing and deformation capabilities, hysteretic curves and skeleton curves, energy dissipation properties (such as damping, ductility, and stiffness), material deformation (such as gypsum, concrete, and steel bars), and fracture characteristics of the typical samples of the (dense-column) latticed concrete gypsum composite panel. The experiment on Q-1 was described as follows. When the sample was pulled over a distance of 0.72 mm, 45° fine, inclined cracks appeared below the initial horizontal crack at the foot of the composite panel. As the load was increased, both inclined and horizontal cracks were on the increase. When the sample was pulled over a distance of 3.28 mm, a lot of dense 45° inclined cracks which differed in length appeared in the middle and lower parts of the composite panel. Some of the cracks in the middle part of the composite panel intersected, causing the gypsum board to peel slightly. As the load applied to the composite panel was increased, the outside steel bars of the panel yielded, and the principal inclined crack gradually took shape. When the sample was pulled over a distance of 11.50 mm, inclined cracks formed a large piece of net in the middle part of the composite panel, the gypsum board peeled seriously, and the concrete at the foot of the composite panel was cracked and crushed, as shown in Figure 10a. The experiment was ended when the composite panel fractured seriously.

The cyclic experimental process revealed that the three composite panel samples underwent four stages: the elastic stage when the load was initially applied to the panel, the elastic-plastic stage when the panel yielded, and the fracture stage when the displacement and deformation of the panel was great while the load applied decreased gradually. The sample was approximately in a square shape and exhibited shear fracture. The final fracture of the samples shown in Figure 10 were primarily reflected by the cracking and crushing of the concrete at the foot of the panel samples (Q-1 and Q-3) or the occurring of the 45° principal inclined crack at the foot of the sample (Q-2).

#### 4.2.2. Hysteretic Characteristics

**Figure 11:**
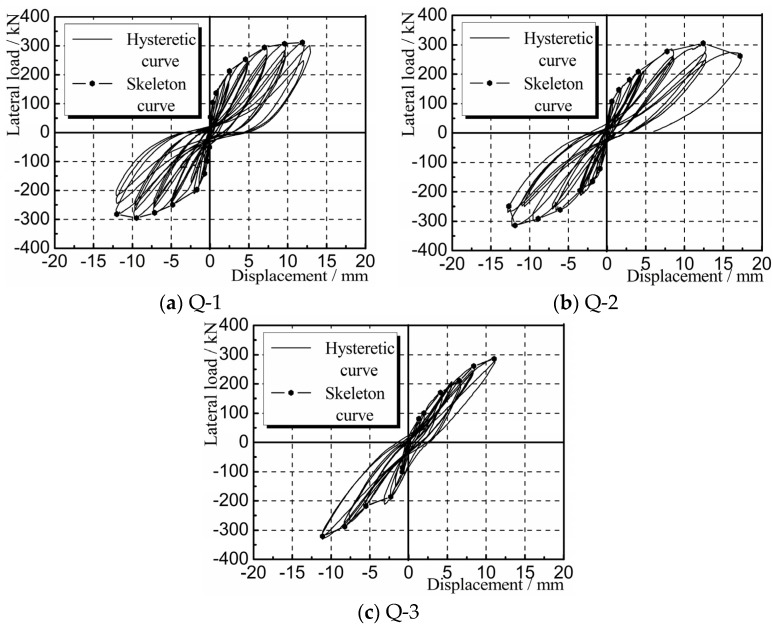
Hysteretic and skeleton curves (**a**) Q-1; (**b**) Q-2; (**c**) Q-3.

Hysteretic curves are the lateral load-displacement curves for the composite panel under cyclic loading. Figure 11 shows the hysteretic and skeleton curves for the panel samples Q-1, Q-2, and Q-3 obtained through experimental measurement. It can be seen that before the composite panel cracked, the load-displacement curves generally showed a linear change. The hysteresis loops initially took a fusiform shape, with no significant change in stiffness, indicating that the samples were in the elastic stage. After the composite panel cracked and yielded, the areas surrounded by the hysteresis loops increased significantly, indicating that the energy dissipation capacity of the samples increased gradually. The shapes of the hysteresis loops produced a “pinch effect”, namely the gradual conversion of the hysteresis loops from a “fusiform” shape to a reversed “S” shape. The stiffness degenerated significantly, indicating that the samples were in the elastic-plastic stage. The hysteretic curves were generally full, indicating that the panel had strong energy dissipation capacity.

#### 4.2.3. Analysis of Material Strain

**Figure 12:**
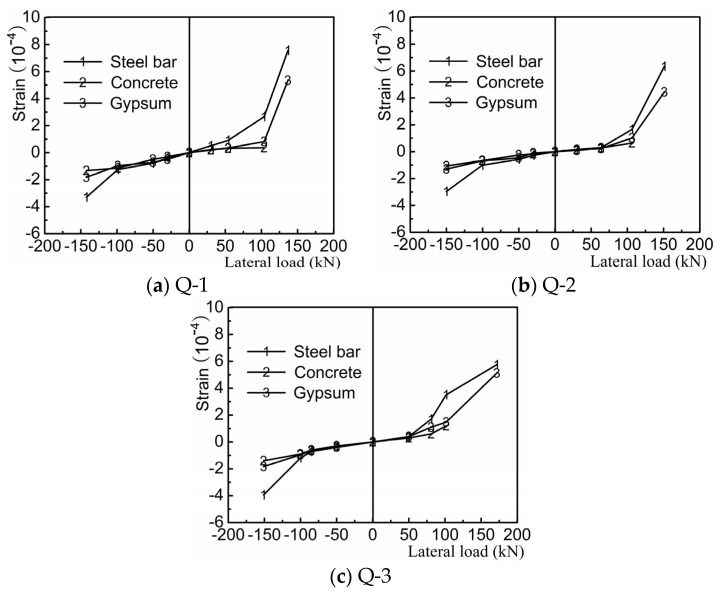
Lateral forces-material strain curves (**a**) Q-1; (**b**) Q-2; (**c**) Q-3.

Material strain is analyzed at the foot same position of three samples outermost steel bars. Materials mainly include steel, concrete and gypsum board. The lateral forces-material strain curves are shown in Figure 12. According to estimates, observing the cracks appearance of wallboard or partial failure of concrete strain gauges and other methods, samples initiation cracks were found basically in control loading of the fourth stage. Therefore, lateral forces-material strain curves only gave key data points' results of the previous four stages. Strain variation width of the three materials is very small, when key positions tensioning effect before the concrete cracked. Strains of steel bars and gypsum board appeared a sudden increase phenomenon after the concrete cracked. Concrete quit his job after the cracking. The force is assumed by other materials at the same time. Instead, the strain sudden increase is not obvious, when key positions compression effect before the concrete cracked. This result illustrates strain variation width plasterboard are largely consistent between concrete and other materials (steel bars and gypsum board).

## 5. Analysis of Finite Element Calculation and Experimental Results

### 5.1. Calculation Results of Elastic Constants

According to the test results of related materials in Section 4.1 and the method for obtaining the values of shear moduli of materials defined in the *Code for Design of Concrete Structures* (GB50010-2010) [19], 40% of the corresponding elastic moduli could be used as the shear moduli of the concrete and gypsum board. The experimental results of the materials were substituted into the relevant equations in Section 3 to obtain the equivalent elastic constants shown in Table 1.

**Table 1:** Equivalent elastic constants.

Equivalent Elastic Constants	Panel Sub-Element I	Panel Sub-Element II	Secondary Homogenous Element
E_x_ (N/mm^2^)	22,248.41	22,248.41	22,248.41
E_y_ (N/mm^2^)	20,816.53	22,248.41	21,622.17
G_xy_ (N/mm^2^)	8326.61	8899.36	8658.20
*ν_xy_*	0.197	0.196	0.196
40%E_x_ (N/mm^2^)	8899.36	8899.36	8899.36
Error (%)	6.43	0	2.71
40%E_y_ (N/mm^2^)	8326.61	8899.36	8648.87
Error (%)	0	0	0.11

An error analysis was performed of the shear constants shown in Table 1 and the values obtained according to the method defined in the code. The errors were generally within 5%, which meets engineering requirements. In this way, equivalence was established between the composite panel with complex material properties and a simple homogenous panel made of a single material with material properties that can be easily determined for ease of further calculation. In the following, a finite element numerical simulation was carried out of typical components to test the feasibility of the simplified calculation model.

### 5.2. Finite Element Simulation

#### 5.2.1. Constitutive Relation of Materials

The equivalent homogenous panel material reference model is commonly used in concrete, *i.e.*, multilingual isotropic (MISO) hardening model is suitable for analysis of simulated seismic cyclic loading. The steel bars within the panel were simulated using the ideal elastic-plastic model with the hardening stage, namely the bilinear isotropic (BISO) hardening model [20]. BISO options used in the von-Mises yield criterion. The initial was a small deformation problem for isotropic material [21]. Yield determination is based on whether reaches the yield strength for the outermost load-bearing steel bars of wallboard. In the calculation process, the stress-strain relationship of the material could be either called in the form of the txt format list or input by writing in ANSYS Parametric Design Language (APDL) provided by software. Displacement control load and force convergence criterion is adopted when solve the model [20]. Displacement control is based on the key points corresponding to the displacement of the test obtained skeleton curves. Convergence error can be generally raised to 5%. This can increase the speed of convergence (*i.e.*, the command is CNVTOL). The constitutive relation of the equivalent homogenous material is shown below [22]:

(25) $$\left\{ \begin{array}{ll} {\text{σ}}_{c}\text{=}f_{c}[1-{\left(1-{\text{ε}}_{c}/{\text{ε}}_{0}\right)}^{n}] & {\text{ε}}_{c}\leq {\text{ε}}_{0}\\ {\text{σ}}_{c}\text{=}f_{c}\{1-0.15[({\text{ε}}_{c}-{\text{ε}}_{0})/({\text{ε}}_{cu}-{\text{ε}}_{0})]\} & {\text{ε}}_{0}<{\text{ε}}_{c}\leq {\text{ε}}_{cu} \end{array}\right.$$

where σ*_c_* is the compressive stress; ε*_c_* is the compressive strain; *f_c_* is the design value of compressive strength; ε*_0_* is the corresponding compressive strain of *f_c_*; and ε*_cu_* is the ultimate compressive strain.

The seismic experimental results of the (dense-column) latticed concrete-gypsum composite panel revealed that in the experimental process, the steel bars were primarily in the elastic and yielding stages, without significant hardening and necking phenomena, whose effects could be ignored during calculation. Therefore, the constitutive relation of the steel bar material could be simplified as an ideal elastic-plastic model [23]. σ*_s_* is the yield strain stress; ε*_s_* is the yield strain.

Namely,

(26) $$\left\{ \begin{array}{ll} \text{σ=}E\text{ε} & \text{ε}\leq {\text{ε}}_{s}\\ {\text{σ=σ}}_{s} & \text{ε}>{\text{ε}}_{s} \end{array}\right.$$

#### 5.2.2. Elements and Solving

**Figure 13:**
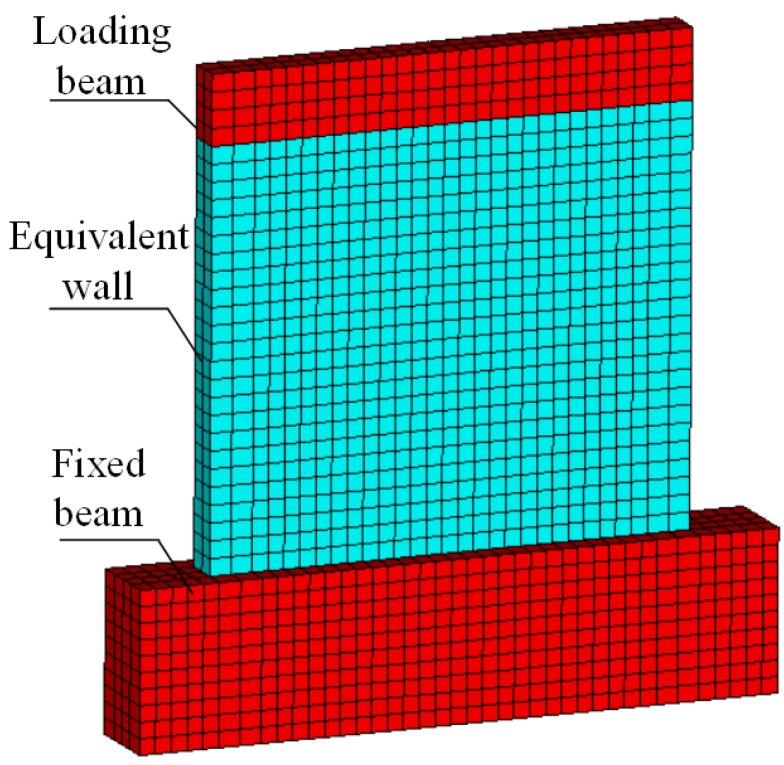
Calculation model for Q-1.

The finite element calculation model for the panel sample shown in Figure 13 consisted of the fixed beam, the panel, the steel bars in the panel, and the loading beam. The equivalent panel material was modeled using the eight-node hexahedral three-dimensional solid element SOLID65, which can process the nonlinearity of materials and simulate materials’ cracking due to tension, crushing due to stress, and plastic deformation. The loading beam and the fixed beam were modeled using the eight-node hexahedral three-dimensional solid model SOLID45. SOLID65 element is established on the basis of SOLID45 element with the addition of special cracking and crushing capabilities. In concrete applications, for example, the solid capability of the element may be used to model the concrete while the rebar capability is available for modeling reinforcement behavior. SOLID65 element can use the Willam–Warnker (*i.e.*, five-parameter failure criterion). Parameters of five-parameter failure criterion can be entered via the command (e.g., TB, CONCR and TBDATA). SOLID65 element may facilitate convergence control through some of the features. For instance, adjusting KEYOPT options; choosing to consider or not consider the additional items of shape functions; choosing separate or integral analysis model; adjusting mesh density; adjusting sub-steps; adopting displacement control load and force convergence criterion, or adopting force control load and displacement convergence criterion; dealing with loading point and support conditions; *etc*.

The vertical load applied to the top of the loading beam and the horizontal seismic load applied to its end could be input as the area loads on the various sides of the element (namely force/area). In the process of simulating the forces applied to the panel, in order to prevent the loading beam and the fixed beam from fracturing earlier than the panel so as to cause a cessation of calculation, the material properties of the two could be appropriately magnified. The steel bars were simulated using the three-dimensional spar element LINK8 [24]. Models were established separately based on the actual dimensions of the panel sample. The steel bars were embedded in the panel material. The steel bars and the homogenous material were assumed to bond firmly and coordinate in deformation. The effects of the bond slip between the equivalent panel material and the steel bars were ignored, and they were treated as being connected through common nodes. The bottom of the fixed beam was fully constrained. The top and end sides of the loading beam were coupled into a rigid region in order to ensure that the loading area did not fracture in the loading process. First, the vertical load was evenly applied to the top of the loading beam in the form of pressure to simulate the vertical load passed down from the structure at the top of the panel. Then, the displacements corresponding to the critical points of the skeleton curves obtained through the seismic experiment on the sample Q-1 were used as control loads to be applied successively to the end of the loading beam. The incremental-iterative method was used for solving.

### 5.3. Result Analysis

**Figure 14:**
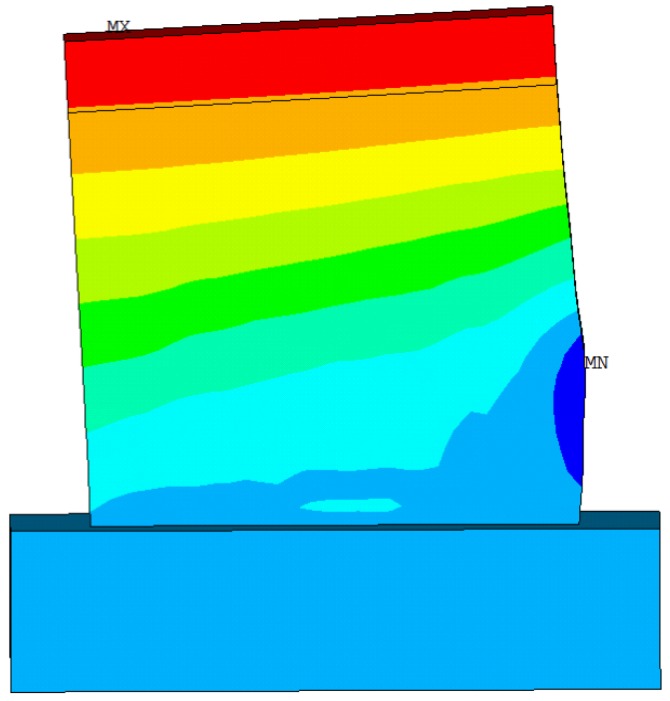
Deformation nephogram for Q-1.

**Figure 15:**
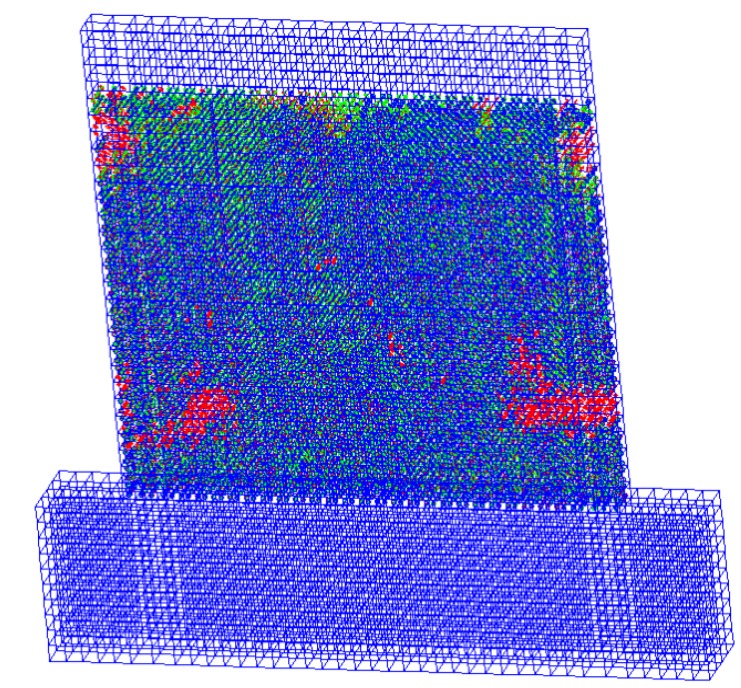
Crack and crush for Q-1.

**Figure 16:**
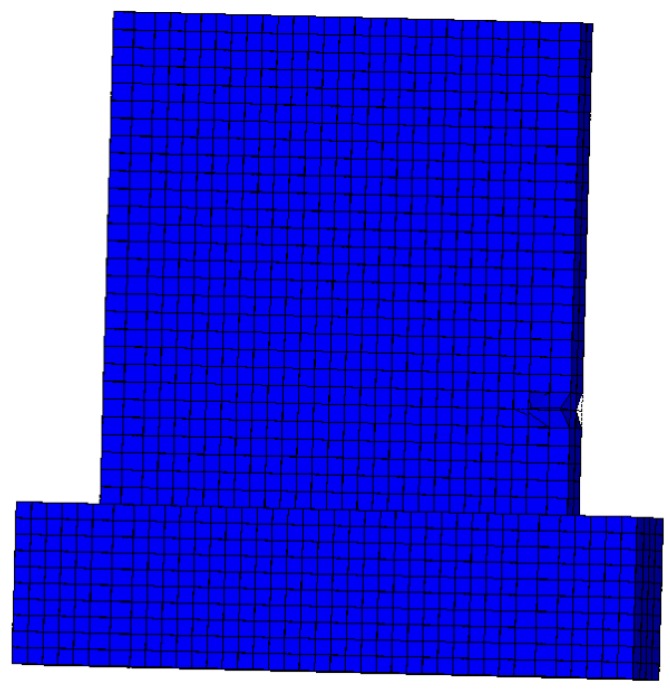
Partial failure for Q-1.

**Figure 17:**
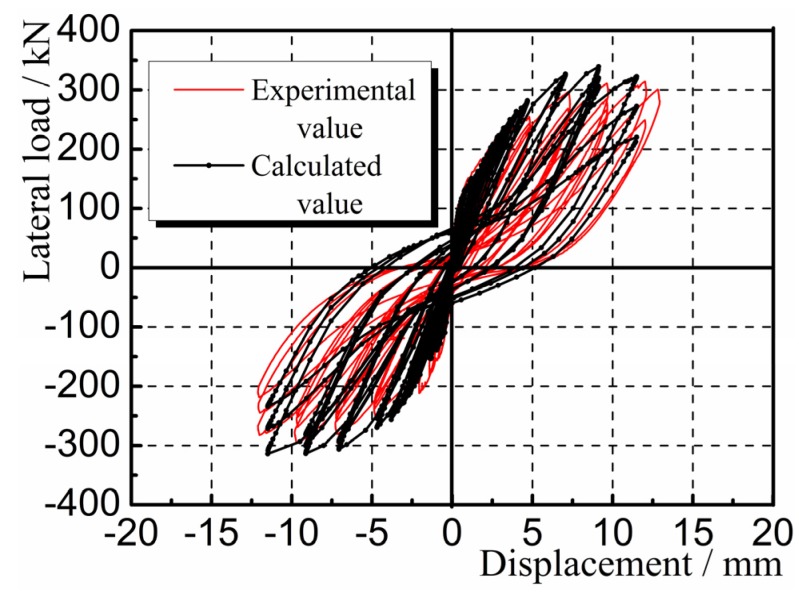
Comparison of hysteretic curves for Q-1.

**Figure 18:**
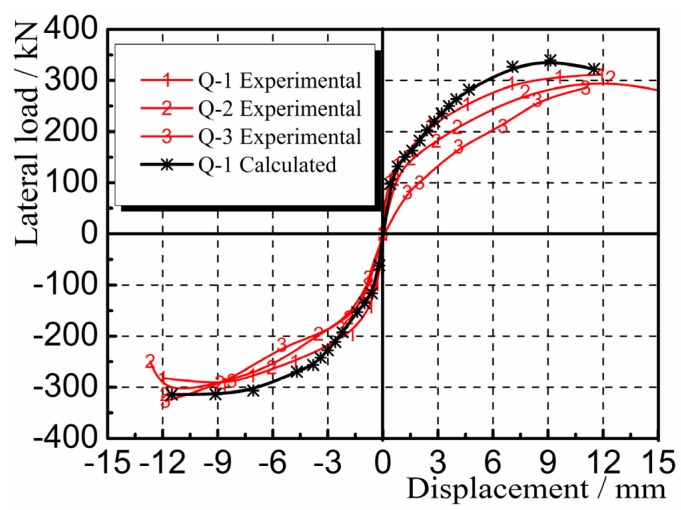
Comparison of skeleton curves.

The deformation nephogram and failure of the sample shown in Figure 14, Figure 15 and Figure 16 were primarily reflected by the failure critical parts at the foot of the panel sample. The main failure formats are the cracking and crushing of the materials or the occurring of the 45° principal inclined crack. As results of the study illustrate, the finite element analysis results are consistent with the main damage phenomena of the test. As shown in Figure 17, the calculated value of the hysteresis loop area of the hysteretic curves for Q-1 was larger than the experimental value, and they looked fuller. This was because the equivalent panel model was not affected by the relative slip between the gypsum, concrete, and steel bars along their interface in the calculation process. The general trends in the skeleton curves depicted in Figure 18 show that the calculated values and experimental values for Q-1 were similar. A comparison of the skeleton curves revealed that the experimental values for the three samples were close to the calculated values for them. As shown in Table 2, comparison of parameters such as bearing capacity, displacement and ductility coefficient revealed that the calculation results were generally consistent with the experimental results, with errors smaller than 5%, indicating that the simplified calculation method for various equivalent elastic constants was reliable and could be used for engineering calculation analysis.

**Table 2:** Comparison of calculation and experimental results.

Value Types	Sample	$P_{cr}$ (kN)	${\text{Δ}}_{cr}$ (mm)	$P_{y}$ (kN)	${\text{Δ}}_{y}$ (mm)	$P_{u}$ (kN)	${\text{Δ}}_{u}$ (mm)	μ
Calculated value	Q-1	61.73	0.72	219.83	3.28	322.41	11.50	3.51
Experimental value	Q-1	60.46	0.84	212.60	3.68	311.85	11.90	3.23
	Q-2	60.11	0.69	206.40	3.50	305.02	12.42	3.55
	Q-3	64.74	0.61	211.50	3.07	327.07	11.85	3.86
Mean of experimental values	$\bar{Q}$	61.77	0.71	210.17	3.42	314.65	12.06	3.55
Error (%)		0.06	1.41	4.60	4.09	2.47	4.64	1.13

Notes: *P_cr_*, *P_y_* and *P_u_* denote the crack load, the yield load and the ultimate load; Δ*_cr_*, Δ*_y_* and Δ*_u_* denote the crack displacement, the yield displacement and the ultimate displacement; and μ denotes the ductility coefficient.

## 6. Conclusions

A detailed theoretical derivation was conducted of the simplified calculation model of the structurally complex (dense-column) latticed concrete gypsum composite panel. The simplified calculation model was used to make a practical calculation of the composite panel, and the calculation results and the experimental results were compared. Finally, the following conclusions were drawn:

(1) The problem of simplified calculation of the composite panel with a complex structure and whose material properties are difficult to determine was addressed, namely the equivalence of the complex composite panel with complex material composition and whose material properties were difficult to determine to a simple homogenous panel whose material properties were easy to determine, facilitating the calculation of the composite panel.

(2) The calculation models of two homogenous composite sub-elements and a secondary homogenous element were established in this paper. Theoretical derivation was conducted of them, respectively, to obtain the formulas for calculating the various equivalent elastic constants. No matter how the basic dimensions (the thickness of the gypsum side panel, the thickness and height of the gypsum partition, and the size of the gypsum cavity) of a composite panel are adjusted, this method is effective, indicating that this method is universal.

(3) Through practical application of the simplified calculation model, the calculation results and the experimental results were compared, validating the correctness and reliability of the simplified calculation model. The application of the simplified calculation model to practical engineering is of great realistic significance.
